# Integrated bioinformatics analysis for conducting a prognostic model and identifying immunotherapeutic targets in gastric cancer

**DOI:** 10.1186/s12859-023-05312-1

**Published:** 2023-05-09

**Authors:** YaLing Liu, Dan Li, Yong Chen, YiJuan Liu, YiJuan Lin, XunRu Huang, Ting Wu, ChengDang Wang, Jian Ding

**Affiliations:** 1 Department of Gastroenterology, the First Affiliated Hospital, Fujian Medical University, Fuzhou, 350005 China; 2 Department of Gastroenterology, National Regional Medical Center, Binhai Campus of the First Affiliated Hospital, Fujian Medical University, Fuzhou, 350212 China; 3 Department of Gastroenterology, Fujian Medical University Union Hospital, Fuzhou, 350001 China

**Keywords:** Gastric cancer, Bioinformatics, Risk score, Prognosis prediction, Immunotherapy

## Abstract

**Background:**

Gastric cancer is the third leading cause of death from cancer worldwide and has a poor prognosis. Practical risk scores and prognostic models for gastric cancer are lacking. While immunotherapy has succeeded in some cancers, few gastric cancer patients benefit from immunotherapy. Immune genes and the tumor microenvironment (TME) are essential for cancer progression and immunotherapy response. However, the roles of immune genes and the tumor microenvironment in immunotherapy remain unclear. The study aimed to construct a prognostic prediction model and identify immunotherapeutic targets for gastric cancer (GC) patients by exploring immune genes and the tumor microenvironment.

**Results:**

An immune-related risk score (IRRS) model, including APOH, RNASE2, F2R, DEFB126, CXCL6, and CXCL3 genes, was constructed for risk stratification. Patients in the low-risk group, which was characterized by elevated tumor mutation burden (TMB) have higher survival rate. The risk level was remarkably correlated with tumor-infiltrating immune cells (TIICs), the immune checkpoint molecule expression, and immunophenoscore (IPS). CXCL3 and CXCL6 were significantly upregulated in gastric cancer tissues compared with normal tissues using the UALCAN database and RT-qPCR. The nomogram showed good calibration and moderate discrimination in predicting overall survival (OS) at 1-, 3-, and 5- year for gastric cancer patients using risk-level and clinical characteristics.

**Conclusion:**

Our findings provided a risk stratification and prognosis prediction tool for gastric cancer patients and further the research into immunotherapy in gastric cancer.

**Supplementary Information:**

The online version contains supplementary material available at 10.1186/s12859-023-05312-1.

## Introduction

Gastric cancer (GC) is an aggressive malignancy with the third-highest mortality rate in the world and an incidence of approximately 5.5% of all new cancer cases worldwide [1]. Approximately one million new cases of GC were diagnosed worldwide in 2020 [2]. Epidemiological studies indicate that the incidence of GC in East Asian countries accounts for about half of all global cases [3]. Many studies have been conducted on the pathogenesis, diagnosis, staging, treatment, and prognosis of GC. However, the prognosis of GC patients remains poor [4]. There are no existing practical tools for risk stratification and prognosis prediction in patients with GC. Treatments for advanced GC patients, regardless of the subtypes, mainly rely on traditional treatment modalities, including surgery, chemotherapy, and radiation therapy. The development of anti-vascular endothelial growth factor receptor 2 (VEGFR2) antibodies and the application of immune checkpoint inhibitors (ICIs) indicates that immunotherapy is a promising treatment modality for GC [5]. However, the complexity of the tumor microenvironment (TME) and tumor immunity hinders the broader application of immunotherapy for GC [6]. Previous studies have explored genes closely related to GC, such as pro-angiogenic genes, suicide genes, et al. [7–9], but few have specifically investigated immune genes as potential targets for GC immunotherapy and prognosis prediction.

To fill this knowledge gap, an immune-related risk score (IRRS) model was conducted using immune genes and applied to stratify GC patients. We systematically investigated the roles of the immune genes in GC immunotherapy and differences in tumor-infiltrating immune cells (TIICs), tumor mutation burden (TMB), immune checkpoint molecule expression level, and immunophenoscore (IPS) in the TME of GC patients in the high- and low- risk groups divided using the risk model. By combining risk level and clinical features, a nomogram was plotted to predict the prognosis of gastric cancer patients.

An innovative prognostic risk score model was proposed based on six immune genes, including APOH, RNASE2, F2R, DEFB126, CXCL6, and CXCL3. Advantages of the model compared with other prognostic models are listed as follows: first: the genes in the model can serve as individual targets and provide better performance when combined than a single factor. Second, the IRRS model was conducted on the basis of six genes that belong to immune genes with unique immune characteristics. Our findings may aid clinicians in risk assessment and prognosis prediction and inform the search for new immunotherapy targets for GC patients.

## Results

### Differential gene expression analysis

**Fig. 1:**
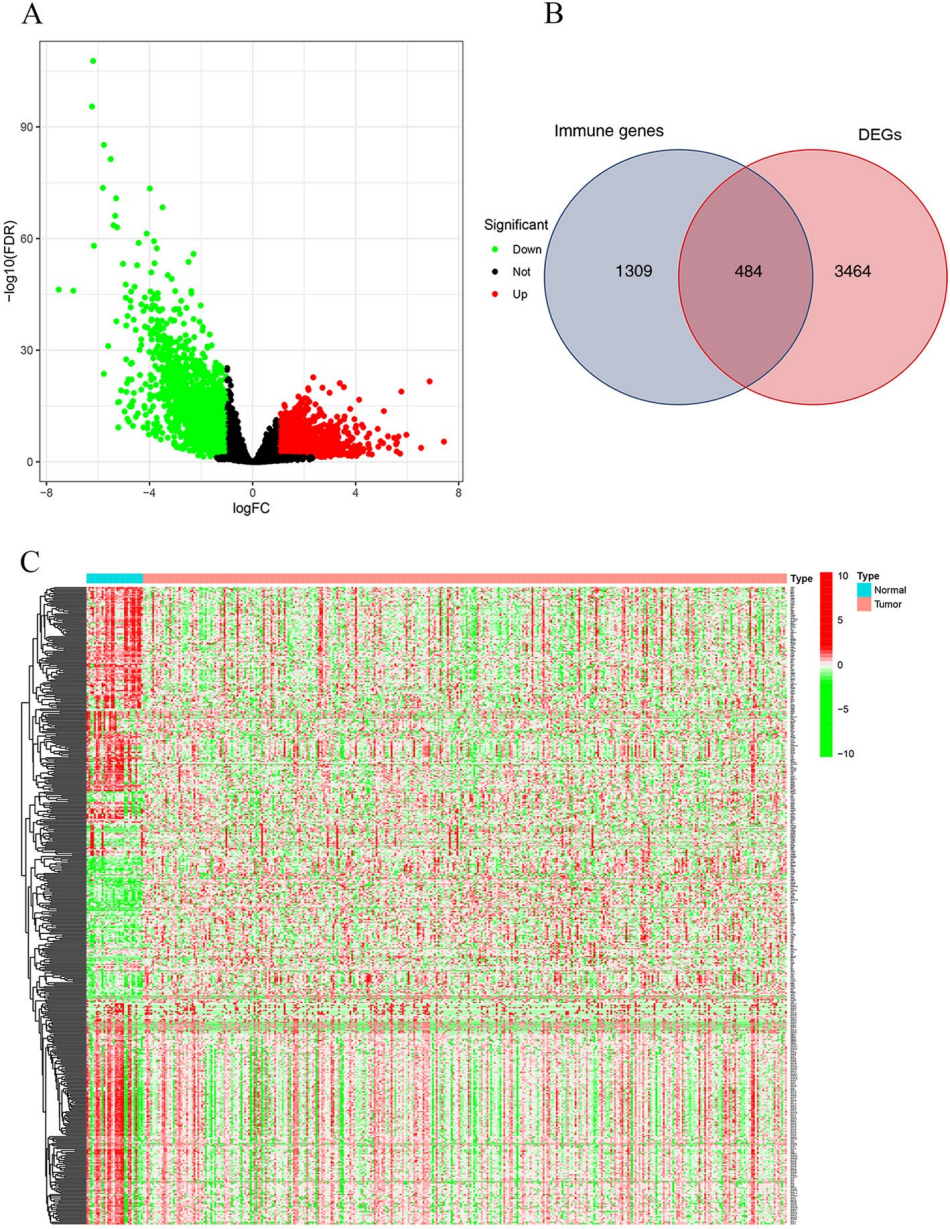
Overview of DEGs in TCGA GC training cohort. **A** An overview of the differential gene expression between GC and normal tissues in TCGA cohorts. **B** Venn diagram showing the intersection of immune genes and DEGs. **C** Heatmap (green: low expression level; red: high expression level) of the DEGs in the tumor samples (red) compared with normal samples (blue)

The volcano plot showed a total of 3948 differentially expressed genes (DEGs) were screened by “edge” and “limma” R packages, including 1693 up-regulated and 2255 down-regulated genes (Fig. 1A). 3948 DEGs were intersected with the 1793 immune genes, and 484 differentially expressed immune genes (DEIGs) were identified (Fig. 1B). Figure 1C shows the heat map of DEGs between GC tumor tissues and normal tissues in TCGA.

### Potential roles of differentially expressed immune genes

**Fig. 2:**
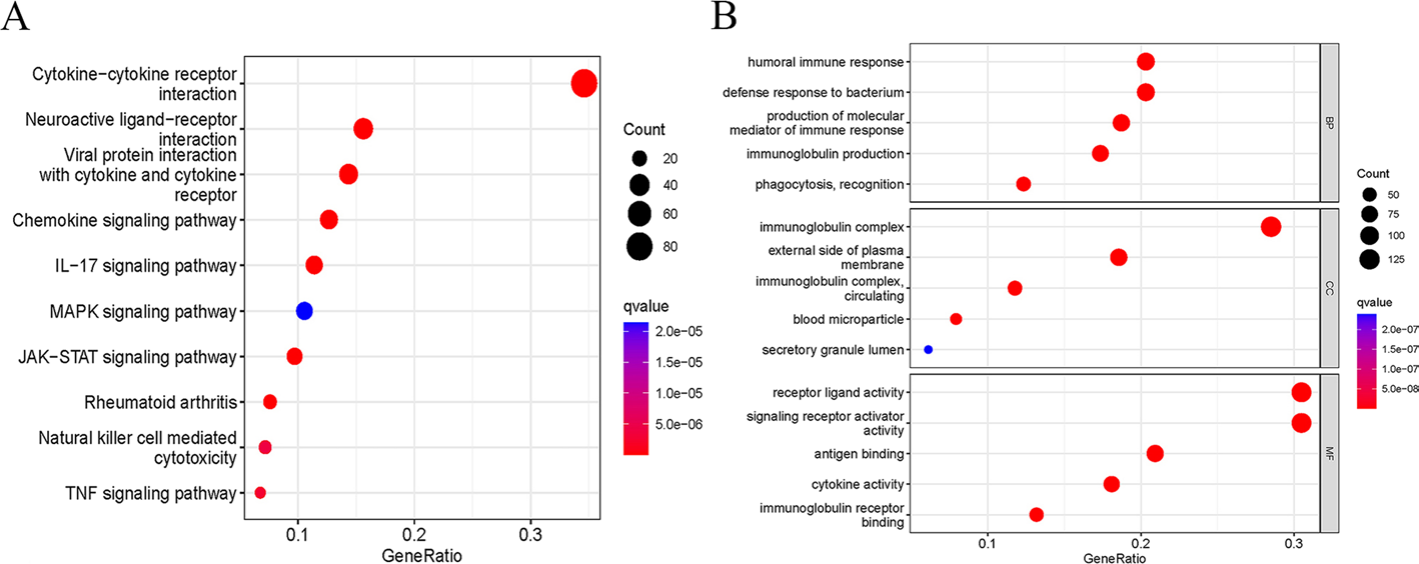
KEGG and GO enrichment analysis of DEIGs

KOBAS-Kyoto Encyclopedia of Genes and Genomes (KEGG) enrichment analysis revealed that cytokine-cytokine receptor interactions were significantly associated with differentially expressed immune genes (DEIGs) in GC **(**Fig. 2A and Additional file 2: Table S1**)**. We also performed Gene Ontology (GO) analysis, which includes biological process (BP), cellular component (CC), and molecular function (MF) categories. In the BP category, DEIGs were related to humoral immune response, defense response to a bacterium, production of molecular, immunoglobulin production, and phagocytosis recognition. For the CC category, DEIGs were related to the immunoglobulin complex, the external side of the plasma membrane, the immunoglobulin complex circulating, blood microparticle, and secretory granule lumen. Receptor ligand activity, signaling receptor activator activity, antigen binding, cytokine activity, and immunoglobulin receptor binding were terms enriched in the MF category (Fig. 2B and Additional file 3: Table S2).

### Identification of immune genes to construct the IRRS model

**Fig. 3:**
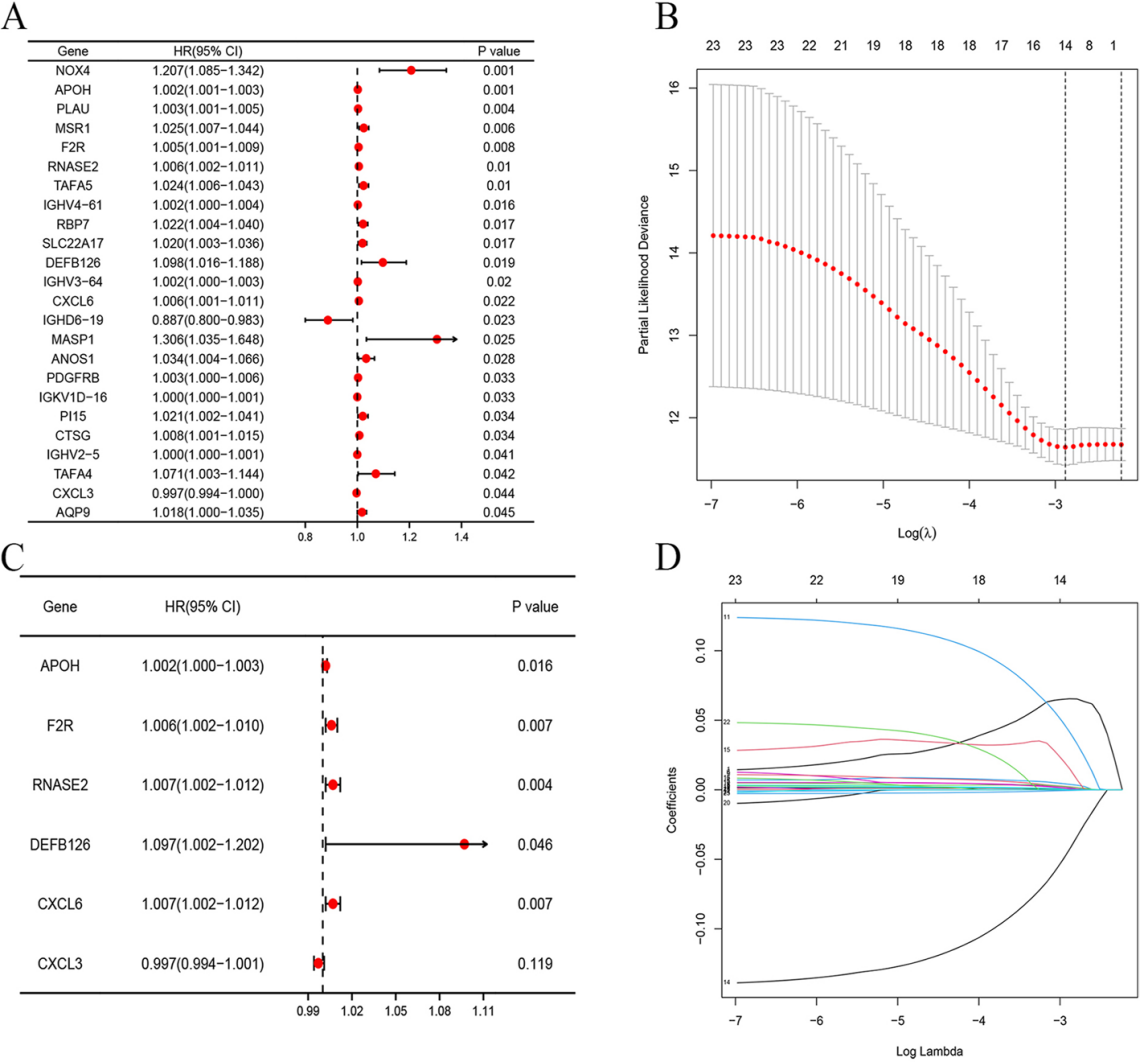
Generation of a gene expression signature for risk assessment on the basis of immune-related clusters. **A** Screening of immune genes related to survival by univariate analysis from TCGA dataset. **B** The optimal parameter (lambda) was selected in the LASSO model; dotted vertical lines were drawn at the optimal values using the minimum criteria. **C** Multivariate Cox regression analyses of overall survival (OS) in TCGA. P-values were obtained by multivariate Cox regression. **D** LASSO coefficient profiles of the candidate OS-related Immune genes with nonzero coefficients determined by the optimal lambda

Univariate Cox regression analysis showed that 24 genes were associated with prognosis (Fig. 3A). LASSO-Cox regression model and the stepwise elimination method on the basis of multivariate Cox regression analysis of 24 genes were performed to identify the most significant Immune genes (*P* < 0.05). To obtain expression data from the GEO dataset to validate the model, we used six genes, the APOH, RNASE2, F2R, DEFB126, CXCL6, and CXCL3 genes, to construct the IRRS model (Fig. 3B–D).

### Establishment and validation of the immune risk score model

**Fig. 4:**
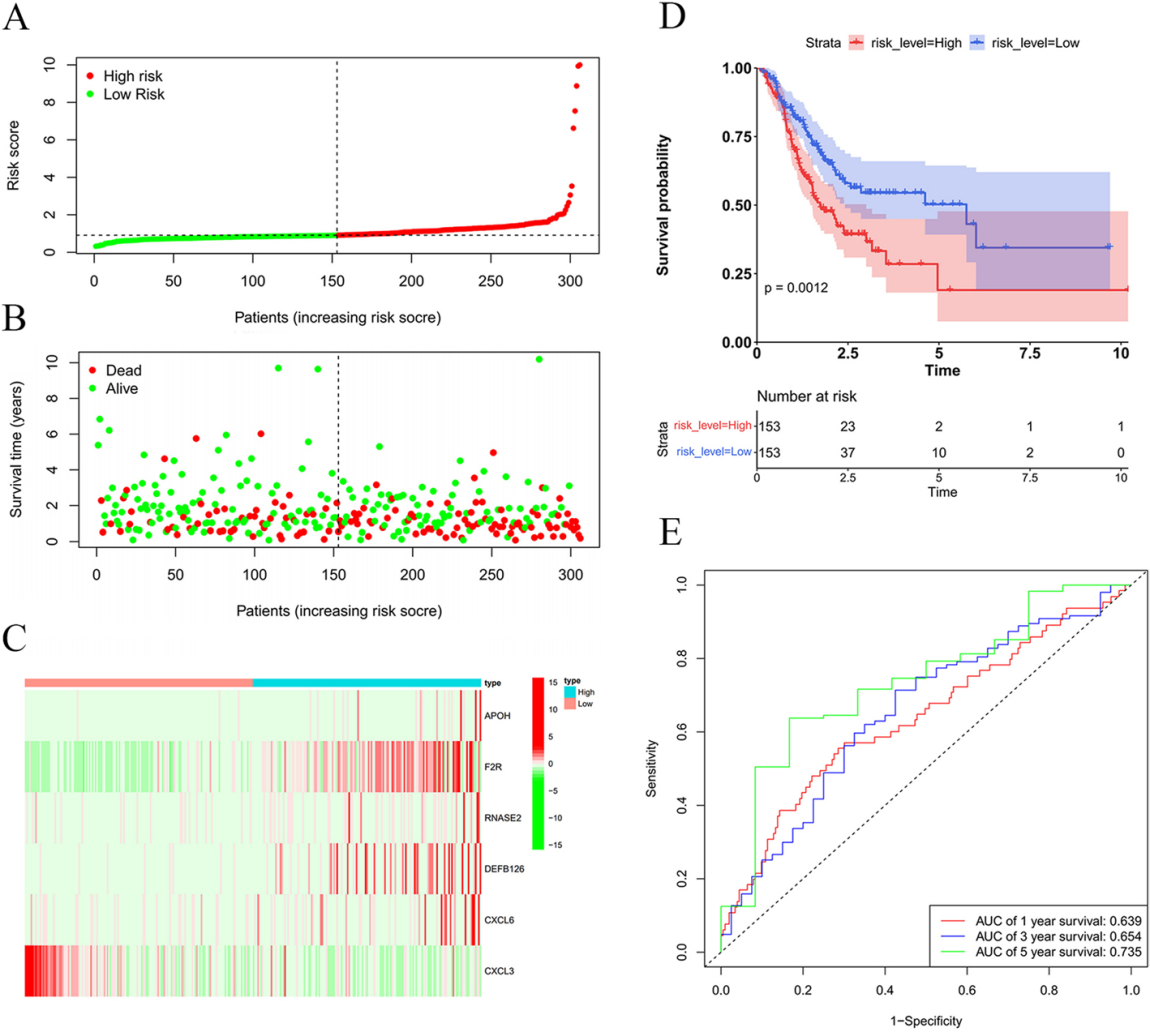
Risk score analyses of GC patients in TCGA using the IRRS model. **A** Distribution of risk scores per patient. **B** Relationships between survival status and survival times of GC patients ranked by risk. The black dotted line represents the median cut-off point used to divide patients into low- and high-risk groups. **C** Heatmap of the six-IRG expression profile. Red to green indicates decreasing expression level, from high to low. **D** Survival analysis of the predictive model. The Kaplan–Meier curves for the high- and low-risk are shown on the top; the number of living patients in the high- and low-risk groups over time (year) are shown on the bottom. **E** The receiver operating characteristic (ROC) curves of predictive models at 1-, 3-, and 5- year. Red represents 1- year, blue represents 3- year, and green represents 5- year

**Fig. 5:**
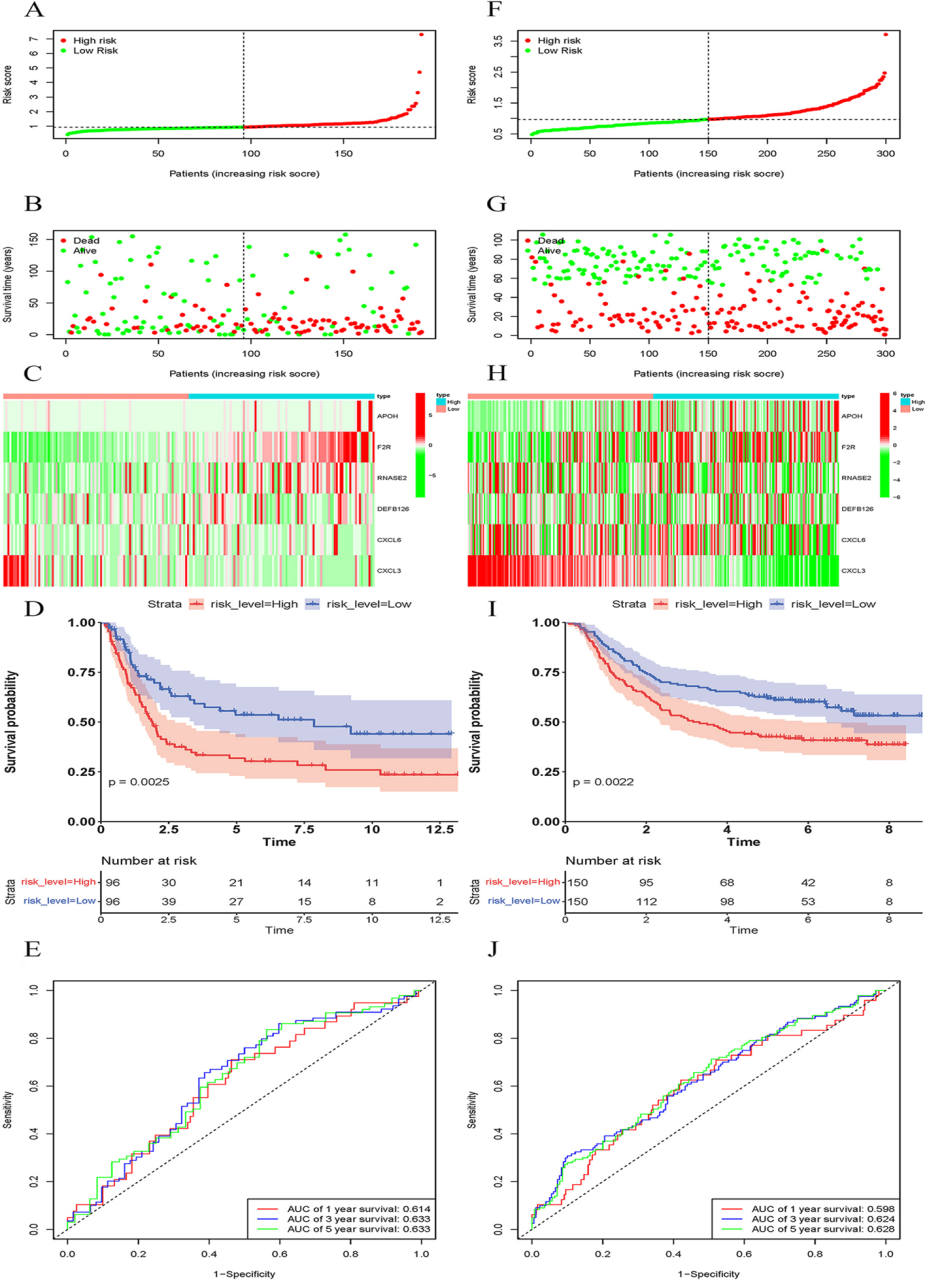
External validation of the risk model using expression data from the GEO database as validation. Patients with lower risk scores showed better OS than those with higher risk scores in **A–E** GSE15459 and **F–J** GSE62254

Enrolled patients were classified into the high- or low-risk group using the calculated median risk score. The risk score formula was constructed according to a linear combination of the expression levels weighted with the regression coefficients from the multivariate Cox regression analysis: Risk score = 0.002 × expression of APOH + 0.006 × expression of F2R + 0.007 × expression of RNASE2 + 0.093 × expression of DEFB126 + 0.007 × expression of CXCL6-0.003 × expression of CXCL3. The distribution of patients with risk scores from low to high is shown in Fig. 4A. We found that the prognosis of GC patients was worse with increased risk score (Fig. 4B). The expression levels of five genes in the IRRS model, except for the CXCL3 gene, were all higher in the high-risk group (Fig. 4C). Kaplan–Meier survival curve showed a survival advantage of the low-risk group over the high-risk group (*P* < 0.05) (Fig. 4D). The area under the curve (AUC) values ranged from 0.639 to 0.735 (Fig. 4E). The model was externally validated with data from the GEO database, and the results were the same as TCGA data, suggesting the excellent predictive capacity of the risk model (Fig. 5A–J).

### Expression levels of the six immune genes

**Fig. 6:**
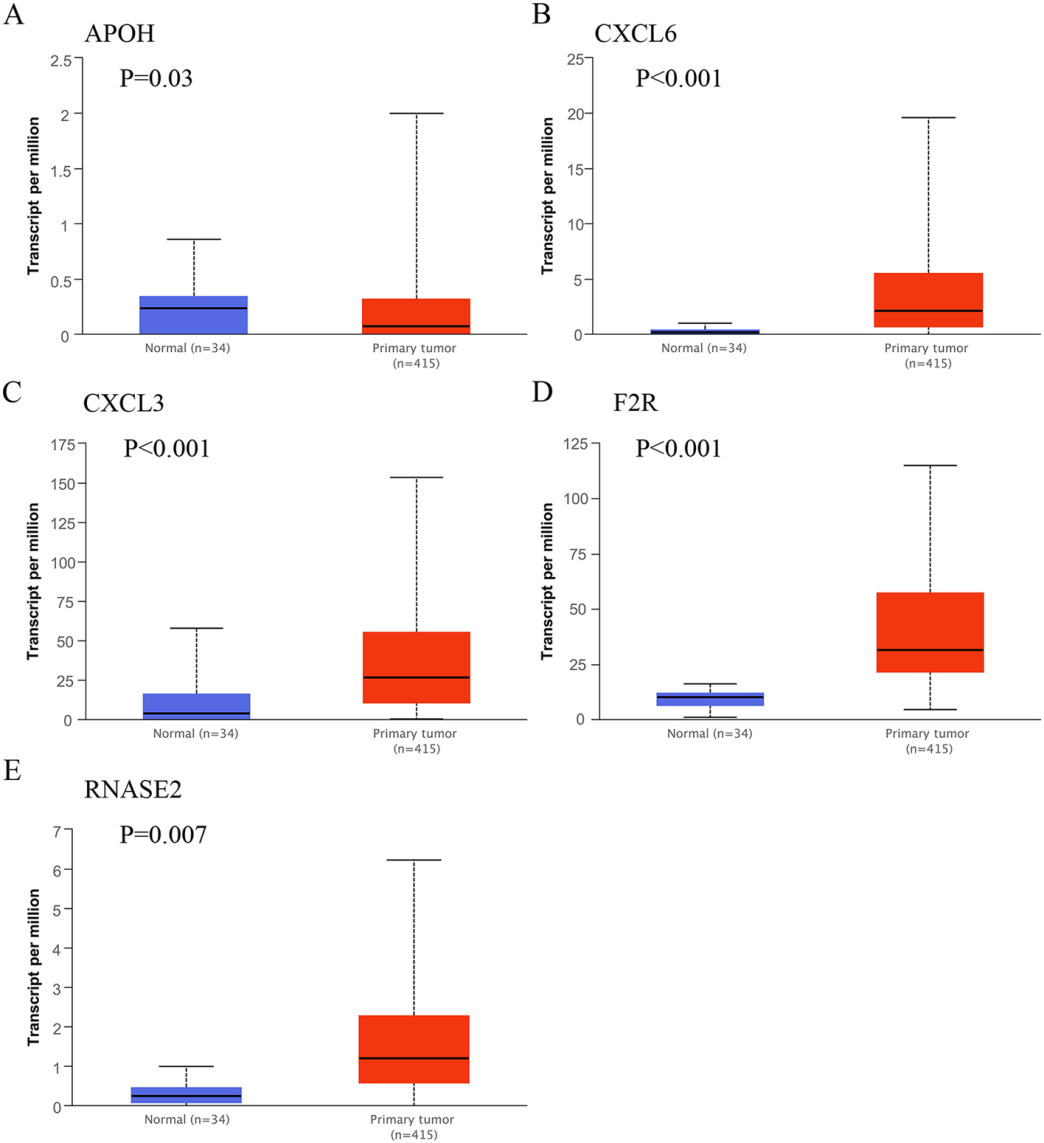
Box plots show the mRNA levels of the five immune genes in primary tumor tissues and normal gastric tissues

**Fig. 7:**
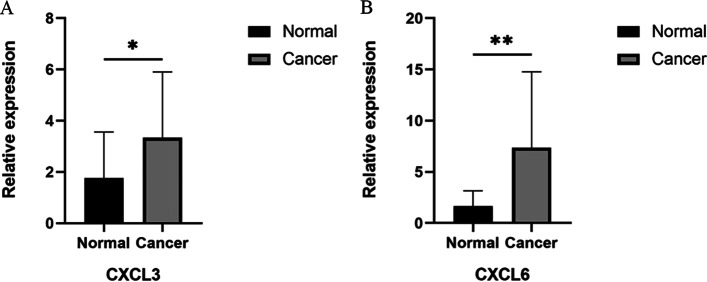
The mRNA levels of CXCL3 and CXCL6 in seventeen pairs of GC tissues and their paired adjacent normal tissues were measured by RT-qPCR (paired *t*-test, * *P* < 0.05; ** *P* < 0.01). **A** CXCL3 and **B** CXCL6

Four immune genes, including CXCL3, CXCL6, F2R, and RNASE2, were upregulated in GC compared to normal tissues in the UALCAN database (Fig. 6). RT-qPCR results showed that tumor tissues exhibited significantly higher expression levels of CXCL3 and CXCL6 mRNAs compared with matched normal tissues, which were in line with the results from the UALCAN database (Fig. 7). The expressions of APOH, DEFB126, RNASE2, and F2R detected by RT-qPCR were shown in Additional file 1: Figure S1.

### Immune cells identification and survival analysis

**Fig. 8:**
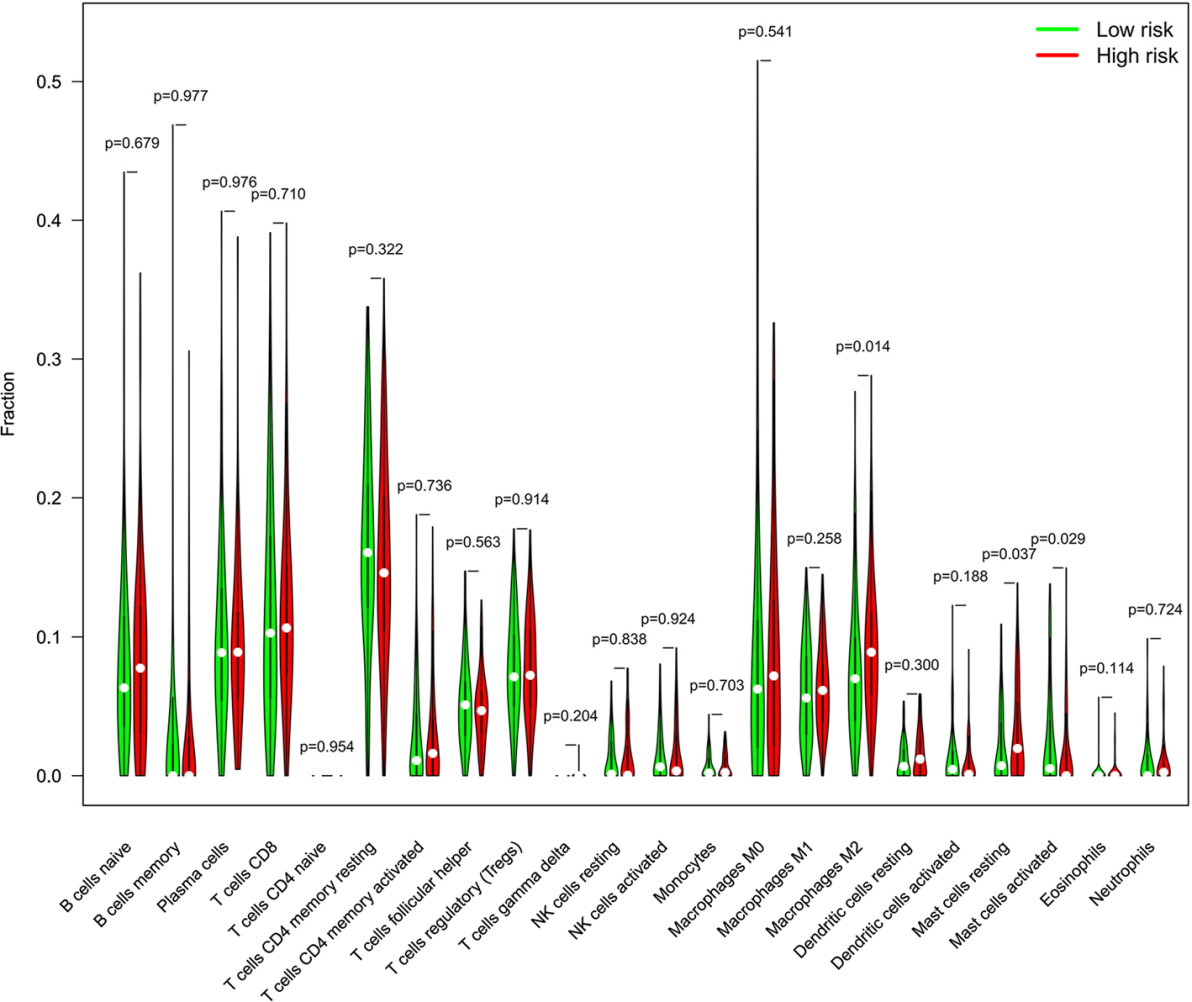
Analysis of TIICs in high- and low-risk groups

The CIBERSORT algorithm [10] was used to estimate the contents of 22 types of immune cells in each sample. The violin diagram shows that the infiltration levels of immune cells, including M2 macrophages and mast cells, were higher in the high-risk group than in the low-risk group, which was statistically significant (*P* < 0.05) (Fig. 8).

### The expression of immune checkpoint molecules in high- and low- risk groups

**Fig. 9:**
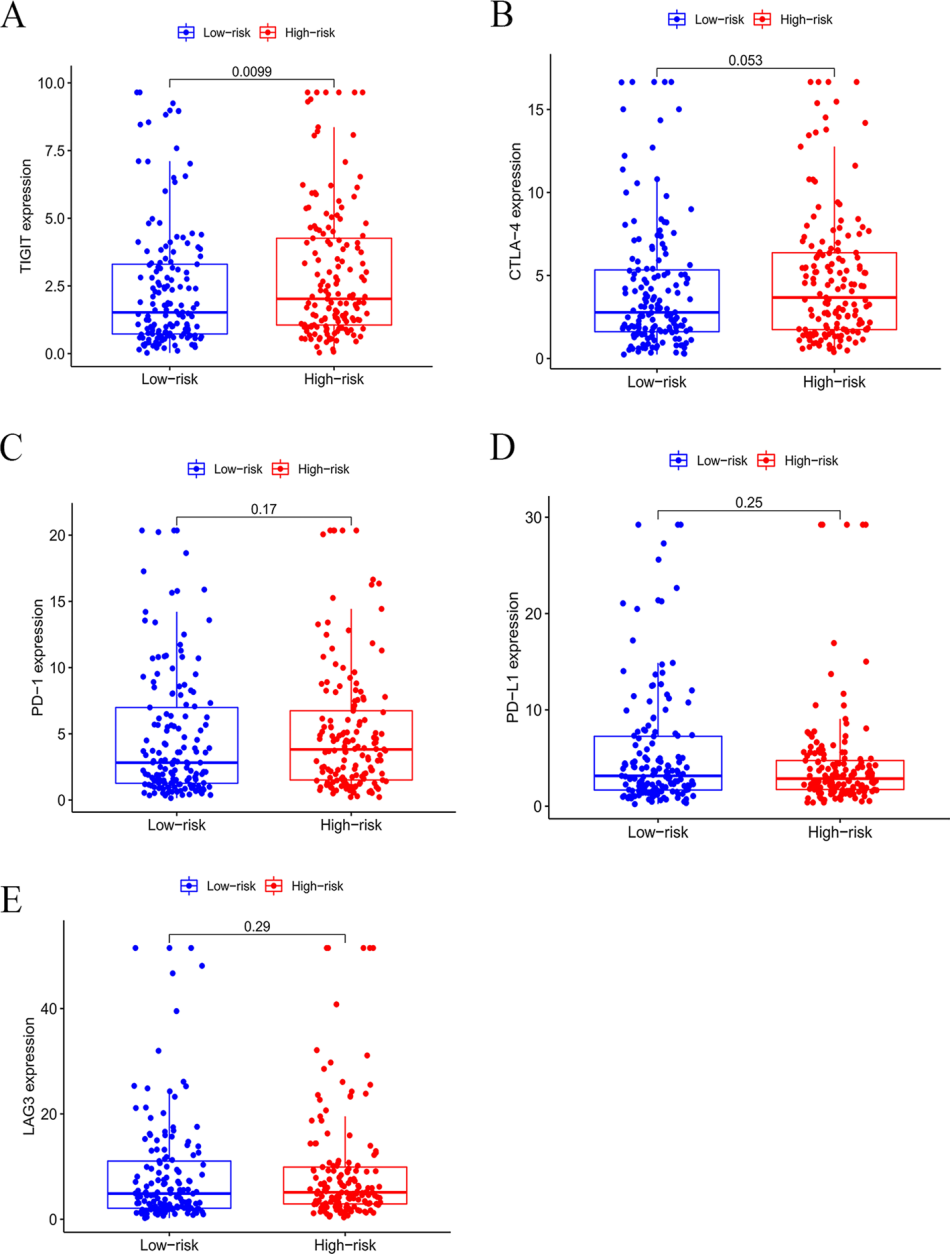
Analysis of immune checkpoint molecule expression levels. **A–E** Differences in expression of five immune checkpoint molecules, TIGIT, CTLA4, PD1, PD-L1, and LAG3, between low- and high-risk groups

We compared the expression of five immune checkpoint molecules (TIGIT, CTLA4, PD1, PD-L1, and LAG3) between high- and low-risk groups. The results indicated TIGIT was significantly upregulated in the high-risk group (Fig. 9A). There were higher expressions of CTLA4, PD1, and LAG3 in the high-risk group, but these differences did not meet statistical significance (Fig. 9B, C, E). Figure 9D showed a trend toward a decreased level of PD-L1 in the high-risk group, but the difference also did not reach statistical significance.

### Immunophenoscores in accordance with the expression of CTLA4 and PD1

**Fig. 10:**
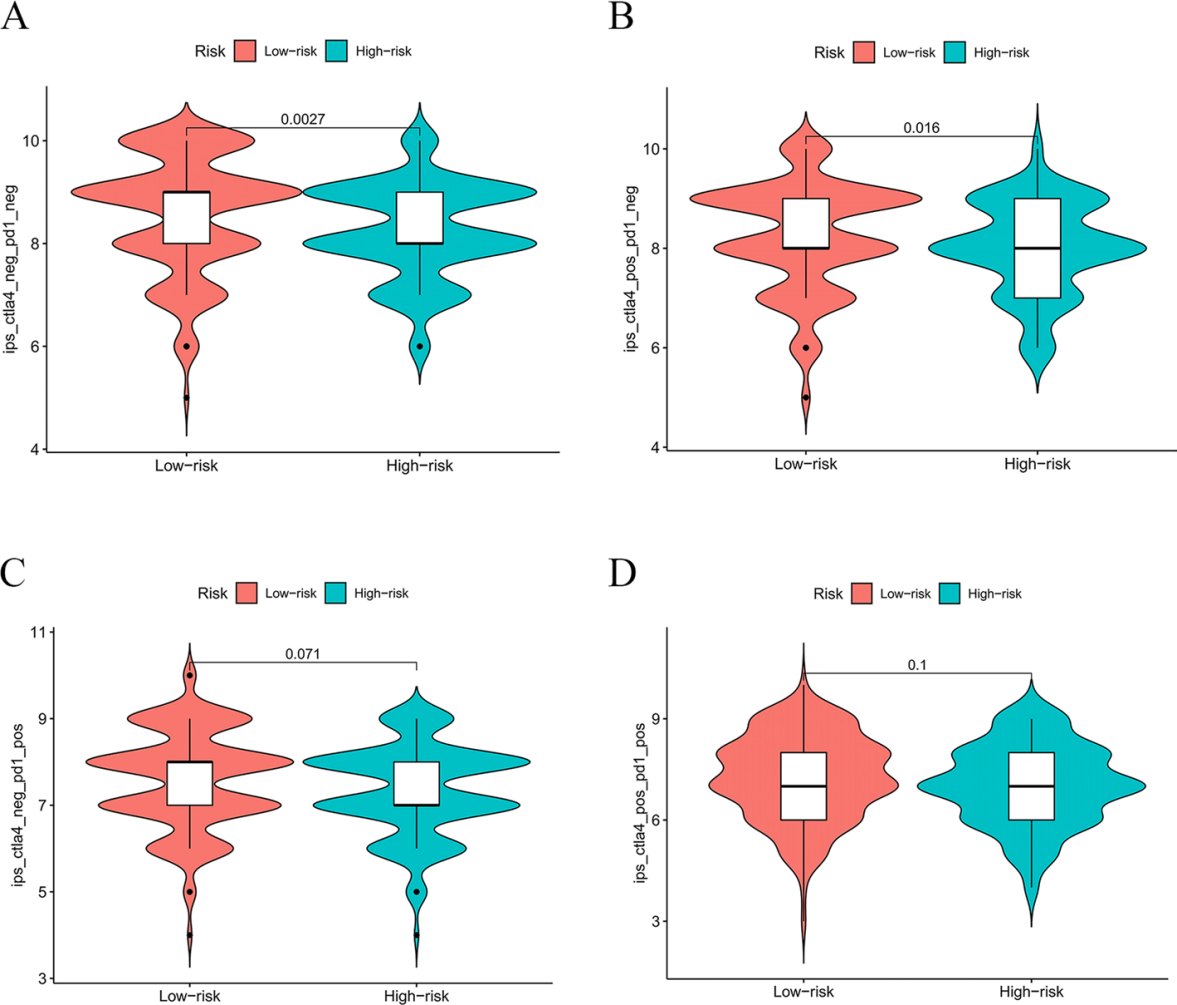
Analysis of IPS in low- and high-risk groups. IPS calculated by expression of CTLA4 and PD1 **A** ctla4_neg_pd1_neg **B** ctla4_pos_pd1_neg **C** ctla4_neg_pd1_pos **D** ctla4_pos_pd1_pos

IPS is a quantitative index that evaluates the effectiveness of checkpoint inhibitors. As shown in Fig. 10, we compared the IPS of patients in low- and high-risk groups in accordance with the expression of CTLA4 and PD1. The IPS of patients with CTLA4-negative/PD1-negative and CTLA4-positive/PD1-negative in the low-risk group significantly differ from that of patients in the high-risk group (Fig. 10A, B). There was no difference between IPS of patients with CTLA4-negative/PD1-positive and CTLA4-positive/PD1-positive in the low- and high-risk groups (Fig. 10C, D).

### TMB in low- and high-risk groups

**Fig. 11:**
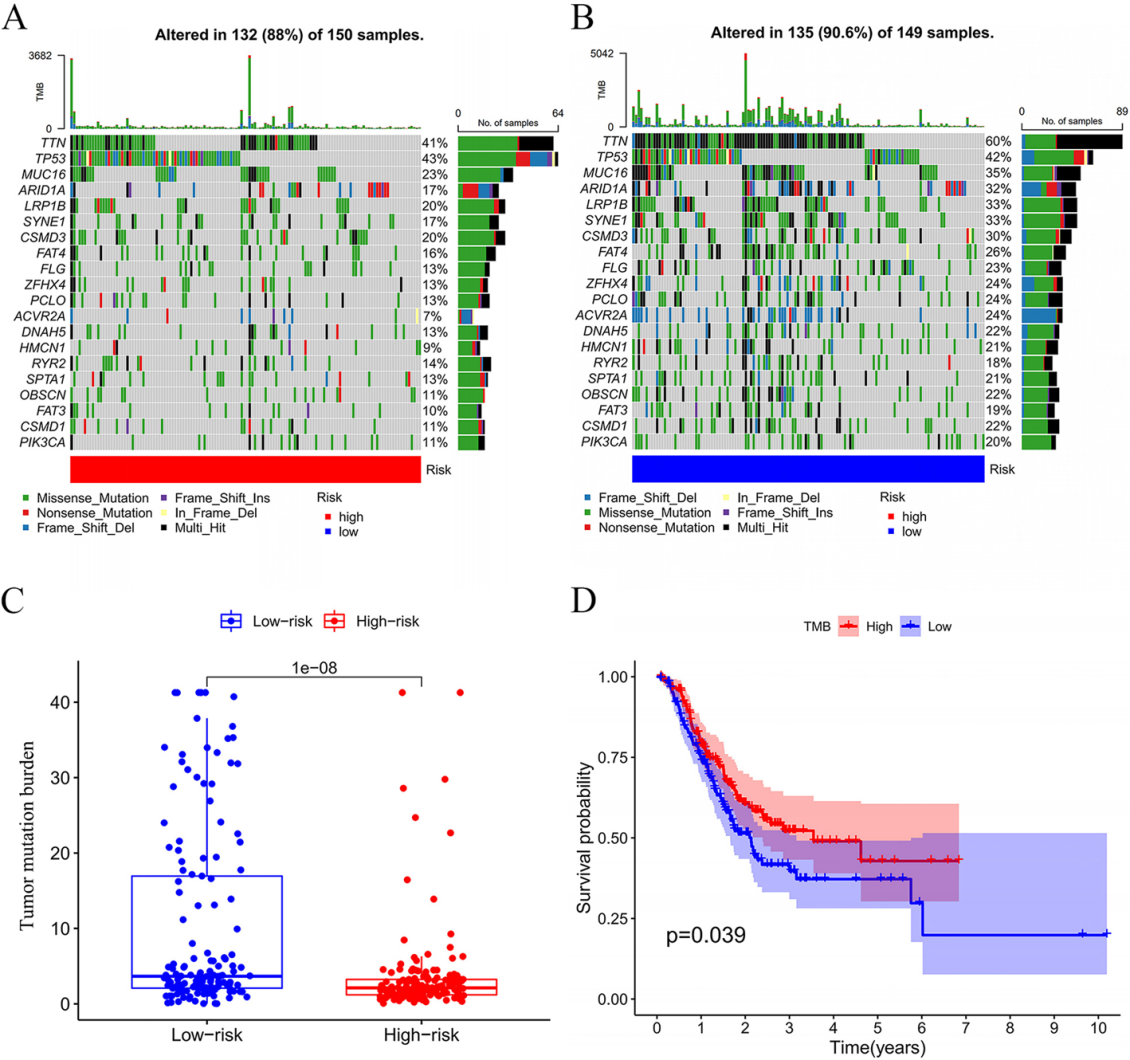
Analysis of gene mutation in high- and low-risk groups. **A**–**B** The landscape of mutation profiles in high- and low-risk groups. The annotations below colors indicate different mutation types. The bar plot on top shows the mutation burden. The numbers on the right represent the mutation frequency. **C** Tumor mutation burden in high- and low-risk groups. **D** Association of tumor mutation burden with survival

The waterfall diagram reveals the integration status of somatic mutations in TCGA GC patients. The numbers of somatic mutations of TTN, TP53, MUC16, LRP1B, CSMD3, SYNE1, ARID1A, and FAT4 gene were higher in low-risk group (Fig. 11A, B). TMB was higher in the low-risk group than  the high-risk group (Fig. 11C). Kaplan–Meier analysis showed that patients with high TMB had a better prognosis (Fig. 11D).

### Exploration of alterations of the six immune genes

**Fig. 12:**
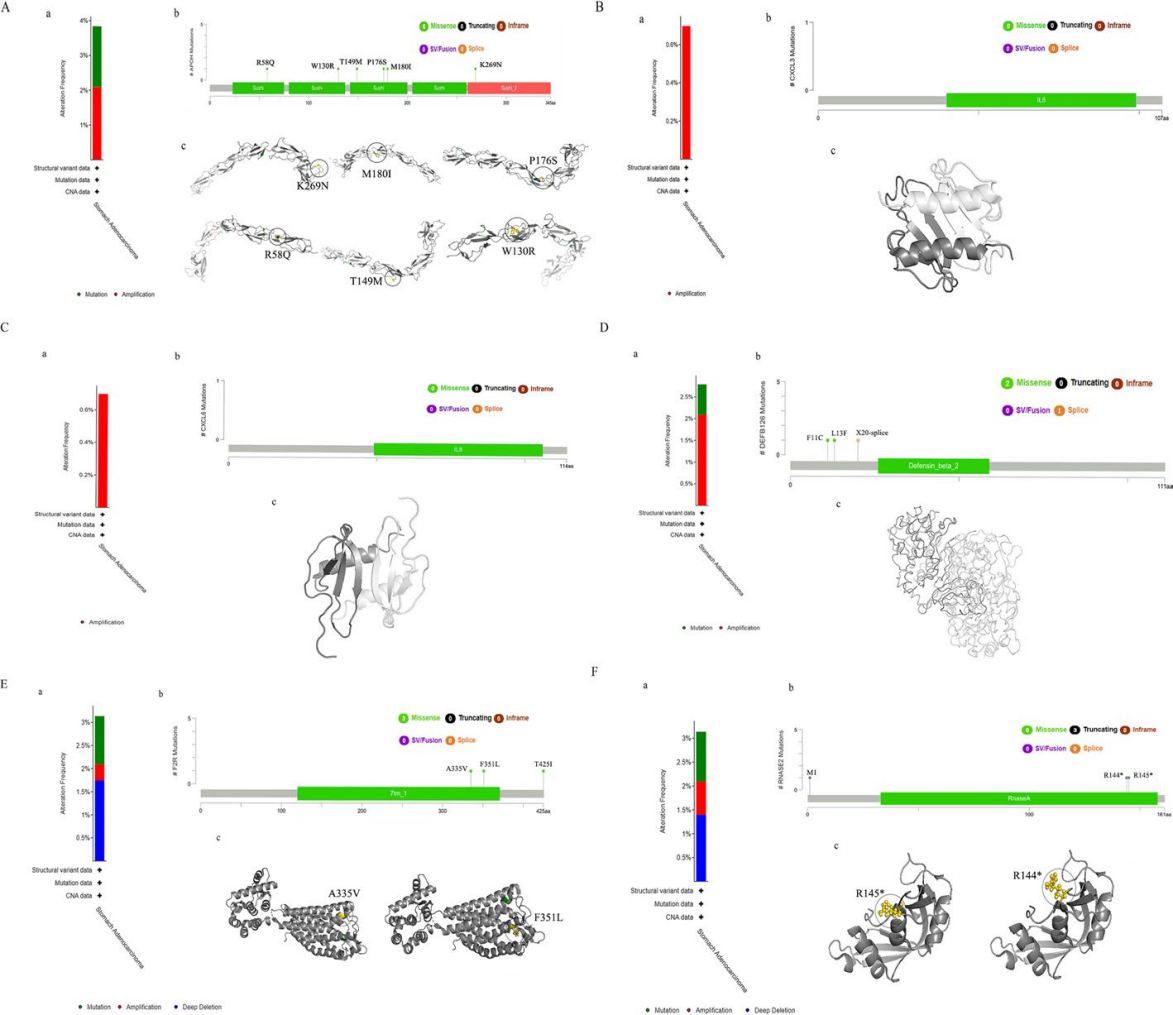
Mutation status of the six immune genes. **a **The alteration frequency with mutation type, **b** mutation site, and **c** mutation site in the 3D structure were shown in: **A** APOH, **B** CXCL3, **C** CXCL6, **D** DEFB126, **E** F2R, and **F** RNASE2

Genetic mutations are key mechanisms underlying tumorigenesis. The genes’ structural variant data, mutation data, and copy number alteration (CNA) data are shown in Fig. 12A, B, C, D, E, F-a. We identified the locations of the mutations within the gene and acquired the mutated site visualized on the three-dimensional (3D) structure of proteins (Fig. 12A, B, C, D, E, F-b, c). We then examined the relationship between specific genetic alterations and the survival of GC patients (Additional file 1: Figure S2A, B, C, D, E, F). Some sporadic mutation sites were seen in APOH, RNASE2, and F2R genes. And no significant association of specific gene mutations with survival was found. Six genes are as shown: **(A)** APOH, **(B)** CXCL3, **(C)** CXCL6, **(D)** DEFB126, **(E)** F2R, and **(F)** RNASE2.

### Development of a nomogram to predict survival of GC patients

**Fig. 13:**
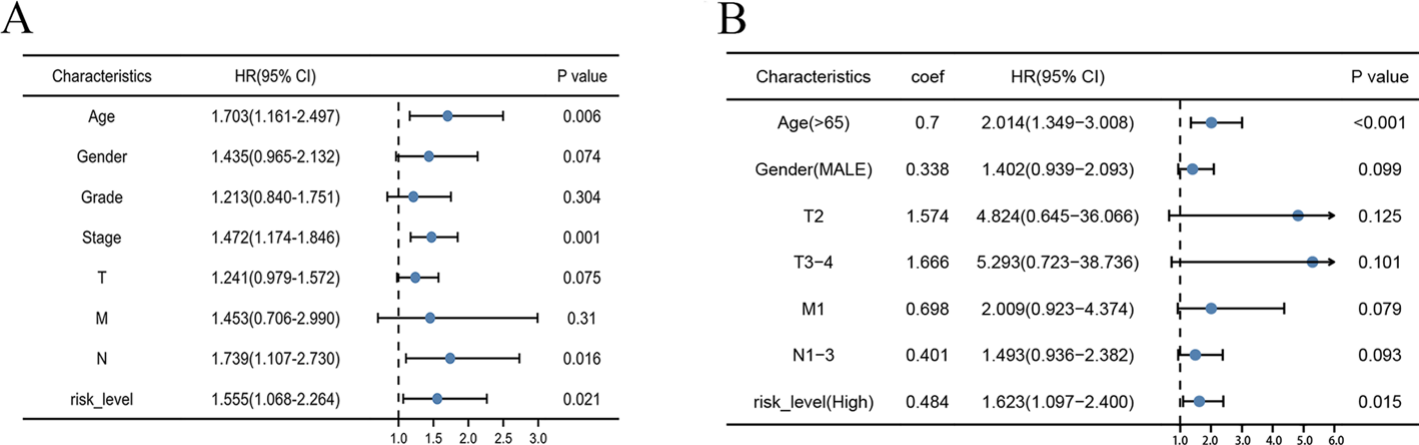
Detailed information on the specific variables involved in the final prognostic model. **A** Screening of OS-related clinical variables by univariate analysis in the GC cohort. **B** Determination of the final variables by multivariate analysis on the basis of the backward stepwise variable selection with the Akaike information criterion (AIC). *P* < 0.05 indicates statistical significance

**Fig. 14:**
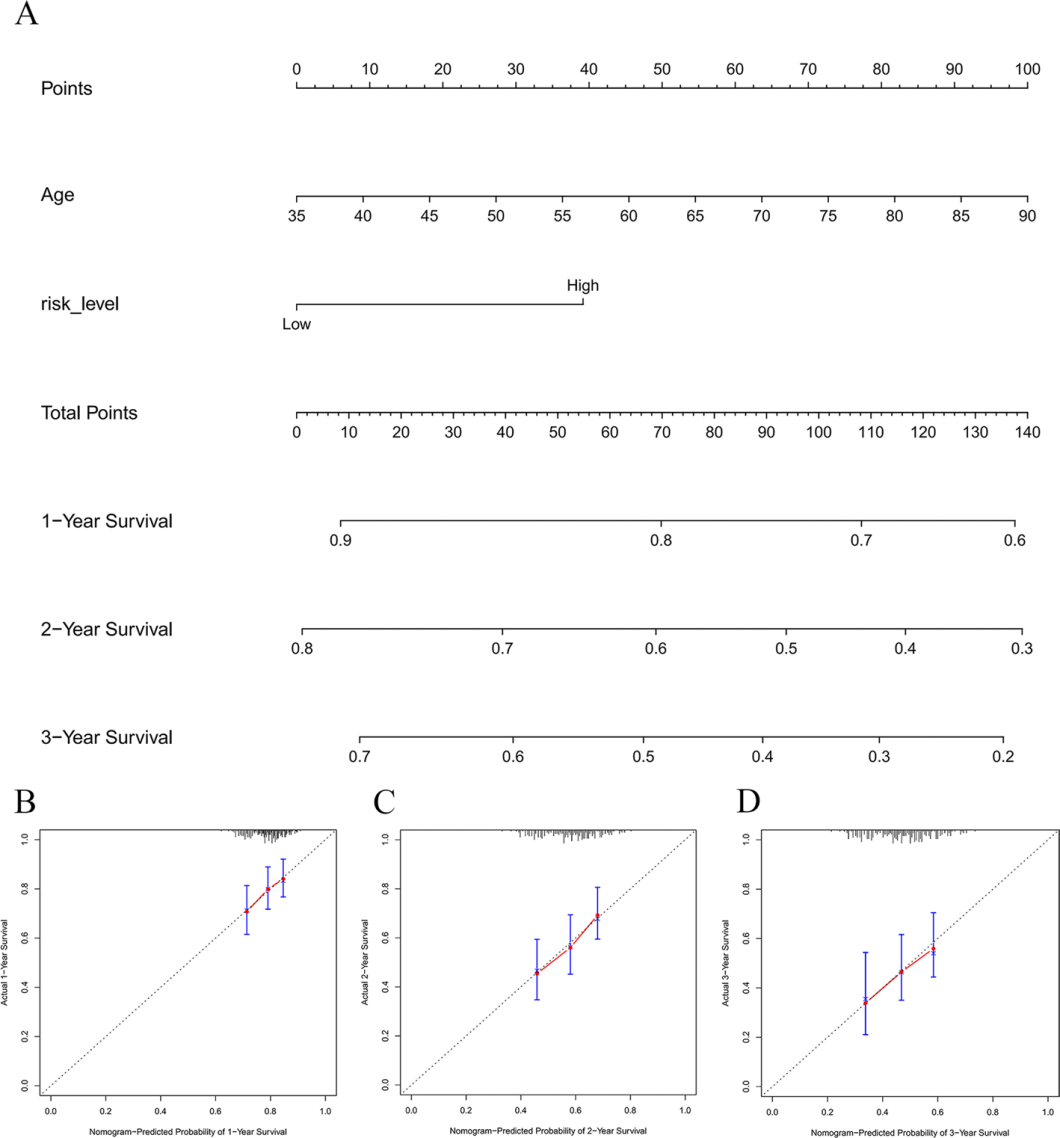
A nomogram diagram considering risk-level and clinical characteristics for predicting the individualized survival probability of GC patients. **A** Nomogram for predicting 1-, 3-, and 5-year OS for GC patients in TCGA cohort. **B–D** Calibration curves of nomogram in terms of the agreement between predicted and observed 1-, 3-, and 5-year outcomes. The 45° dashed line represents the ideal performance; the red lines show the actual performance

The results of univariate Cox regression analysis showed that age, stage, N-stage, and risk level are survival-related factors (Fig. 13A). From the results of multivariate Cox analysis (Fig. 13B), we ultimately chose risk-level, age, gender, T-stage, N-stage, and M-stage to develop a nomogram after the gradual optimization of Akaike information criterion (AIC) values (Fig. 14A). The result indicated that a higher total score corresponded with a shorter survival time of the patients. The calibration curves of the nomogram for the survival probability at 1, 3, or 5 years suggest a great clinical application value (Fig. 14B, C, D). The C-index of nomogram is 0.656, suggesting good accuracy in predicting the survival probability for GC patients.

## Discussion

GC has a high incidence and mortality rate globally [11], and there is an urgent need to develop practical tools for risk stratification and prognosis prediction for GC patients. Widespread attention has been devoted to the research of immunotherapy in cancer management. Immunotherapy has been successfully used to treat various cancers, including lung and breast cancer [12] [13]. Immunotherapy may be an effective treatment for GC patients [4]. Here we identified a novel IRRS model with immune genes for risk stratification. We investigated the expression level and mutation status of genes in the model and TIICs, TMB, immune checkpoint molecule expression levels, and IPS in the TME of patients in high- and low-risk groups. We developed a nomogram with risk level and clinical characteristics for predicting the survival of GC patients.

Increasing evidence has shown that a single gene feature is vulnerable to multiple factors. In this study, we identified 3948 DEGs, including 484 Immune genes, by analyzing data from the TCGA database. Through KEGG and GO enrichment analysis, the DEIGs were mainly enriched in cytokine-cytokine receptor interaction, humoral immune response, immunoglobulin complex, and receptor ligand activity pathways, which may be the main signaling pathways affecting the prognosis of GC patients. Univariate and multivariate Cox regression analyses and the LASSO-Cox regression model identified a combination of six immune genes (APOH, RNASE2, F2R, DEFB126, CXCL6, and CXCL3 genes) to construct the IRRS model. A nomogram is a convenient-to-use tool for individualized prognosis prediction in clinical practice that can help develop a follow-up and treatment plan suitable for patients. In this study, age, gender, T-stage, N-stage, and M-stage were independent prognostic factors for GC patients. Risk level and clinical characteristics were incorporated into a nomogram. The IRRS model had a powerful capacity for risk stratification in GC patients. Immune genes in the model and immune checkpoints in TME may be targets for the immunotherapy of GC. The nomogram, combining clinical features and risk level, demonstrates good sensitivity and specificity for prognosis prediction.

These findings have guiding significance in formulating follow-up strategies for GC patients and improving the effect of GC immunotherapy. Immune genes are associated with the occurrence, development, and metastasis of multiple cancers, such as non-small cell lung cancer, lung squamous cell carcinoma, esophageal cancer, and stomach cancer [14–17], and are essential targets for immunotherapy. Among the six genes identified in our analysis, CXCL3 was the only protective factor. Validation by the UALCAN database and PCR experiments revealed that CXCL3 and CXCL6 were significantly highly expressed in GC tissues. CXC chemokines are a subfamily of chemotactic cytokines with a CXC motif at the N-terminus. CXCL3 acts on the CXCR2 receptor, whereas CXCL6 acts on both CXCR1 and CXCR2 receptors, ultimately resulting in the recruitment of tumor-associated neutrophils and the promotion of tumor angiogenesis [18]. Notably, we found that CXCL3 expression levels gradually decreased as the risk of GC patients increased. This could be explained by a compensatory increase of CXCL3 expression at the early stage of tumorigenesis, thus protecting the body. When some types of cancer cells fail to express CXCL3 highly, the degree of tumor malignancy and the risk of patients are increased. In the cBioPortal database, we found that the mutation type of CXCL3 and CXCL6 in GC was an “amplification” mutation, but its alteration frequency was only approximately 0.7%, indicating that amplification-based mutations were not associated with gene overexpression in tumor tissues; no mutations were detected on the main domains of CXCL3 and CXCL6 genes. CXC chemokines are mainly enriched in chemokine signaling pathways and cytokine-cytokine receptor interaction pathways in renal cancer [19]. Chemokine signaling pathways are vital in various cancers' immune evasion and metastasis [20, 21]. KEGG pathway enrichment analysis indicated that DEIGs are mainly enriched in the cytokine-cytokine receptor interaction and chemokine signaling pathways. Therefore, we hypothesize that the mechanisms of CXCL3 and CXCL6 affecting the progression of GC may be related to these pathways. These data indicate that CXCL3 and CXCL6 may be potential drug therapeutic targets as factors in immune-related signaling pathways in GC. Previous studies showed that CXCL6 enhances the growth and metastases of ESCC cells both in vitro and in vivo [22], and the expression of APOH in hepatocellular carcinoma and colorectal cancer was higher than that in adjacent tissues [23, 24]. The results are consistent with our findings in GC. Other evidence suggests that CXCL6 and APOH are potent oncoproteins that promote tumor growth. PCR showed that APOH, RNASE2, F2R, and DEFB126 mRNA were not significantly highly expressed in GC tissues. These observations suggest that CXCL3 and CXCL6 are major contributing factors in the model. No significant differences were observed with the other four genes, possibly due to racial differences in gene expression and insufficient sample size. Nevertheless, this result does not reduce the validity and accuracy of the model.

With the development of cancer vaccines, ICIs, and CAR-T cells, immunotherapy has made breakthroughs in treating cancers such as melanoma, non-small cell lung cancer, and prostate cancer [25–27]. Immunotherapy exerts potent anti-tumor effects by regulating the immune system and inducing long-lasting immune responses and tumor regression in advanced cancer patients [28]. However, drug resistance, immune escape, and unpredictable efficacy in response to immunotherapies are challenges [29]. The TME, composed of extracellular matrix, T cells, B cells, neutrophils, macrophages, and other components, has an essential impact on immunotherapeutic efficacy [30–32]. Interactions between immune cells and tumor cells in the TME determine the elimination and progression of tumors [33]. We examined TIICs in TME of patients categorized using the risk model. M2 macrophages, mast cell activated, and mast cell resting infiltrations were significantly elevated in patients with high-risk scores, indicating that these cells were associated with risk stratification in GC patients. Some possible mechanisms may explain these results. Macrophages act in wound healing and autoimmune diseases by secreting various cytokines and growth factors [34, 35]. Activated macrophages are classified into anti-tumor M1 and pro-tumor M2 types by the action of cytokines such as TGF-β1, IL-4, and IL-13 [36, 37]. Previous studies showed that M2 macrophages in GC tissue promote tumor progression and metastasis, resulting in poor prognosis [38–40]. Our results showing that the high-risk group was highly infiltrated with M2 macrophages provide further evidence of the tumor-promoting functions of M2 macrophages in GC. *Helicobacter pylori* (Hp) also promotes macrophage polarization from M1 macrophages to M2 macrophages [40]. We suspect that the mechanisms of the Hp trigger GC development [41] may be associated with macrophages. Tumor-associated mast cells (TAMCs) play a pro-tumor or anti-tumor role depending on the tumor type, stage of tumor development, and spatial distribution in the tumor tissue [42]. Mast cells are recruited to the TME by cytokines produced by tumor cells such as vascular endothelial growth factors (VEGFs), angiogenic hormone (ANGPT1), CCL2, and CXCL12 chemokines. The cytokines activate specific receptors on the surface of mast cells and play a vital role in the spatial distribution of TAMCs in the TME [43]. One study found that the density of mast cells in the TME is associated with the formation and progression of micro-vascularization in GC patients [44]. Mast cells release VEGF-A, VEGF-C, VEGF-F, CXCL-8, MMP-9, and other factors, which promote tumor angiogenesis. Thus, the tumor-promoting effects of mast cells cannot be neglected in GC development. We speculate that chemokines secreted by tumor cells in the high-risk group may result in high levels of mast cell infiltration. Increased levels of mast cells then promote tumor growth by mediating angiogenesis. The immune cells mentioned above have dual roles in tumorigenesis: as a tumor suppressor and a tumor growth promoter. The results reveal the complexity and heterogeneity of the TME and show the potentially important roles of the immune cells in immunotherapy. Therefore, we suspect that distinct prognostic outcomes for patients with the same GC subtype may be associated with the types of TIICs and the degree of infiltration in TME.

TMB is associated with immunotherapy response in various cancers, including breast cancer, non-small-cell lung cancer, and colorectal cancer [45–48]. We found that patients in the low-risk group were characterized by a higher TMB, and patients with high TMB had longer OS than those with low TMB. This may be because cell mutations generate a variety of neoantigens leading to cancer cells being more susceptible to being recognized and cleared by the immune system after increased antigen diversity. Previous studies found that TMB can predict ICIs’ efficacy. TMB has emerged as a valuable biomarker for identifying patients who will benefit from immunotherapy in melanoma and non-small cell lung cancer [49, 50]. Considering that neoantigens are more likely to appear on HLA molecules on the surface of tumor cells in patients with TMB-Hi [51, 52], it is rational to hypothesize that patients with TMB-Hi may be more receptive to continuous immunotherapy because of increased antigenic diversity.

Over the past decade, many cancer patients have derived significant clinical benefits from immunotherapy targeting immune checkpoint molecules. In the study, we obtained two meaningful findings. First, the IPS of patients with CTLA4-positive/PD1-negative in the low-risk group was significantly higher than that of patients with high risk. This result reveals that patients with CTLA4-positive/PD1-negative respond better to immunotherapy. The mechanism may be as follows: PD-1 inhibits T lymphocyte’s immune surveillance, resulting in tumor cells’ immune escape [53]. Therefore, immunotherapy response is significantly improved in PD-1-negative patients. Second, TIGIT expression was elevated in the TME of GC patients in the high-risk group. T cell immunoglobulin and ITIM domain (TIGIT), along with PD-1 and CTLA-4, is an immune checkpoint molecule and a novel ICI receptor. TIGIT expressed by tumor cells and antigen-presenting cells in the TME is critical in limiting innate and adaptive immunity against tumors [54, 55]. These results suggest that anti-CTLA4 inhibitors and anti-TIGIT inhibitors may be promising immunotherapeutic agents for GC.

However, our model should be validated further by performing both animal experiments and drug trials to verify the immunotherapy targets better. These have not only increased the challenges but also made us more motivated to continue exploring.

## Conclusion

Here we present a risk score model based on six immune genes, including APOH, F2R, RNASE2, DEFB126, CXCL3, and CXCL6 genes. Combined with the clinical factors, the model can calculate the survival rate of individual patients at 1-, 3-, and 5-year and inform individualized treatment plans and follow-up strategies. Furthermore, CXCL3 and CXCL6 may be new targets for the immunotherapy of GC. The type and degree of TIICs may be related to the prognosis of GC patients. TMB may help to predict the efficacy of immunotherapy in GC patients. Anti-CTLA4 inhibitors and anti-TIGIT inhibitors may be effective immunotherapeutic agents for GC. We hope these results will contribute to furthering the potential application of immunotherapy for GC.

## Materials and methods

### Data acquisition

The RNA-seq and clinical data of 373 samples, including 343 gastric adenomas and adenocarcinoma samples and 30 normal samples, were downloaded from the TCGA database (https://portal.gdc.cancer.gov/) and used as the training set. Additional samples were obtained from GSE62254 (*n* = 300) and GSE15459 (*n* = 192) in the GEO database (http://www.ncbi.nlm.nih.gov/geo) and used as the validation set. Samples in the training set with missing clinical information or survival time less than one month were excluded.

Detailed clinical information is available in Additional file 4: Table S3. The immune genes list was downloaded from the IMMPORT database (https://www.immport.org/). Seventeen pairs of RNA samples of gastric adenocarcinoma and normal tissues were collected from the First Affiliated Hospital of Fujian Medical University, China.

### Gene set enrichment analysis

GO pathway enrichment analysis and KEGG pathway enrichment analysis [56]of DEIGs were performed using the “clusterprofiler [57]”, “org.Hs.eg.db”, and “ggplot2” R packages.

### Identification and verification of the gene signature

The “edge” and “limma” R packages were used to analyze the DEGs with the threshold values of |logFC|> 1 and FDR < 0.05. Differentially expressed genes were intersected with immune gene lists to obtain DEIGs. Univariate and multivariate Cox risk regression analyses and LASSO regression analyses were performed to identify the key Immune genes for conducting IRRS. The UALCAN database [58] (http://ualcan.path.uab.edu/) and qRT-PCR were used to verify genes.

### Construction and prognostic analysis of the IRRS model

The risk score formula was a linear combination of the expression levels weighted with the corresponding regression coefficients derived from multivariate Cox regression analysis as follows: risk score = expression of a gene [1] × corresponding coefficient [1] + expression of a gene [2] × corresponding coefficient [2] + expression of the gene [n] × corresponding coefficient [n] of the gene. The mean score was used for grouping. Kaplan–Meier(K-M) survival curves were plotted to analyze survival. ROC curves and the AUC values were applied to determine prediction efficiency. The data derived from the GSE62254 and GSE15459 in the GEO database, the verification sets, was substituted into the risk score model to validate.

### RNA extraction and quantitative PCR

Total cellular RNA was extracted from human gastric cancer tissue using TRIzol reagent (Invitrogen, Carlsbad, CA, USA) following the manufacturer’s instructions. cDNA synthesis was performed using the PrimeScript RT reagent kit (Takara, Dalian, China). We conducted RT-qPCR assays with SYBR Prime Script RT PCR kit (Takara, Dalian, China). The gene expression levels of candidate mRNAs were normalized to 18srRNA expression levels. The relative quantification of mRNAs was calculated using the 2−ΔΔCT method [59–61]. The sequences of all primers used in this study are provided in Additional file 5: Table S4.

### Exploration of tumor-infiltrating immune cells

The CIBERSORT algorithm was used to analyze the infiltrating immune cells [62]. A violin plot was constructed to visualize the distribution of immune cells of patients in high- and low-risk groups.

### The role of checkpoints expression and IPS in the prediction of immunotherapeutic benefits

Immune checkpoint expression levels were evaluated in the TME of patients in high- and low-risk groups. The IPS data of every patient in the TCGA cohort were downloaded from the TCIA (https://tcia.at/home) [63]. The IPS was calculated using the expression of immune checkpoints, including CTLA-4 and PD-1.

### Calculation of TMB and correlation with prognosis

TMB data were downloaded by the “TCGAbiolinks [64]” R package from the TCGA database. The maf files were read using the “Maftools [65]” R package, and the number of variants in each sample was counted based on the MutSigCV algorithm [66]. The waterfall function within the “Maftools” package was applied to present the mutation landscape.

### Analysis of genetic alterations

Data on alteration frequency, mutated site information, CNA, and 3D protein structure were obtained using the cBioPortal tool [67] (https://www.cbioportal.org/). Survival data, including OS and disease-free survival (DFS), were compared for the patients with or without genetic alterations.

### Development of a nomogram combing risk score and clinical characteristics

We developed a nomogram including the risk level and clinical characteristics. ROC curve analyses and AUC values were used to assess the discriminatory capacity of the model. The calibration curves and a concordance index (C-index) were created to assess the predictive accuracy of the nomogram.

### Statistical analysis

All statistical analyses were performed using R software (version 4.2.0) and GraphPad Prism 9.3 software. *P*-values < 0.05 were regarded as statistically significant.

## Supplementary Information

**Additional file 1. Figure S1:** The mRNA levels of four immune genes. **Figure S2:** Relationships between genetic alterations and survival.

**Additional file 2. Table S1:** KEGG enrichment analysis of DEIGs.

**Additional file 3. Table S2:** GO enrichment analysis of DEIGs.

**Additional file 4.** Detailed clinical information.

**Additional file 5.** Sequences of all primers.

## Data Availability

The raw data could be obtained from online databases including the TCGA database (https://portal.gdc.cancer.gov/), the GEO database (http://www.ncbi.nlm.nih.gov/geo), the IMMPORT database (https://www.immport.org/), the UALCAN database (http://ualcan.path.uab.edu/), the TCIA (https://tcia.at/home) and the cBioPortal tool (https://www.cbioportal.org/) without any restrictions. Further inquiries can be directed to the corresponding author.
