# Supplementary material for: Integrated bioinformatics analysis for conducting a prognostic model and identifying immunotherapeutic targets in gastric cancer
Source: BMC Bioinformatics. 2023 May 9;24:191. doi: 10.1186/s12859-023-05312-1 (PMC10170748; doi:10.1186/s12859-023-05312-1)
Supplement: Supplementary file 1 — Additional file 1. Figure S1: The mRNA levels of four immune genes. Figure S2: Relationships between genetic alterations and survival. [file 12859_2023_5312_MOESM1_ESM.doc]

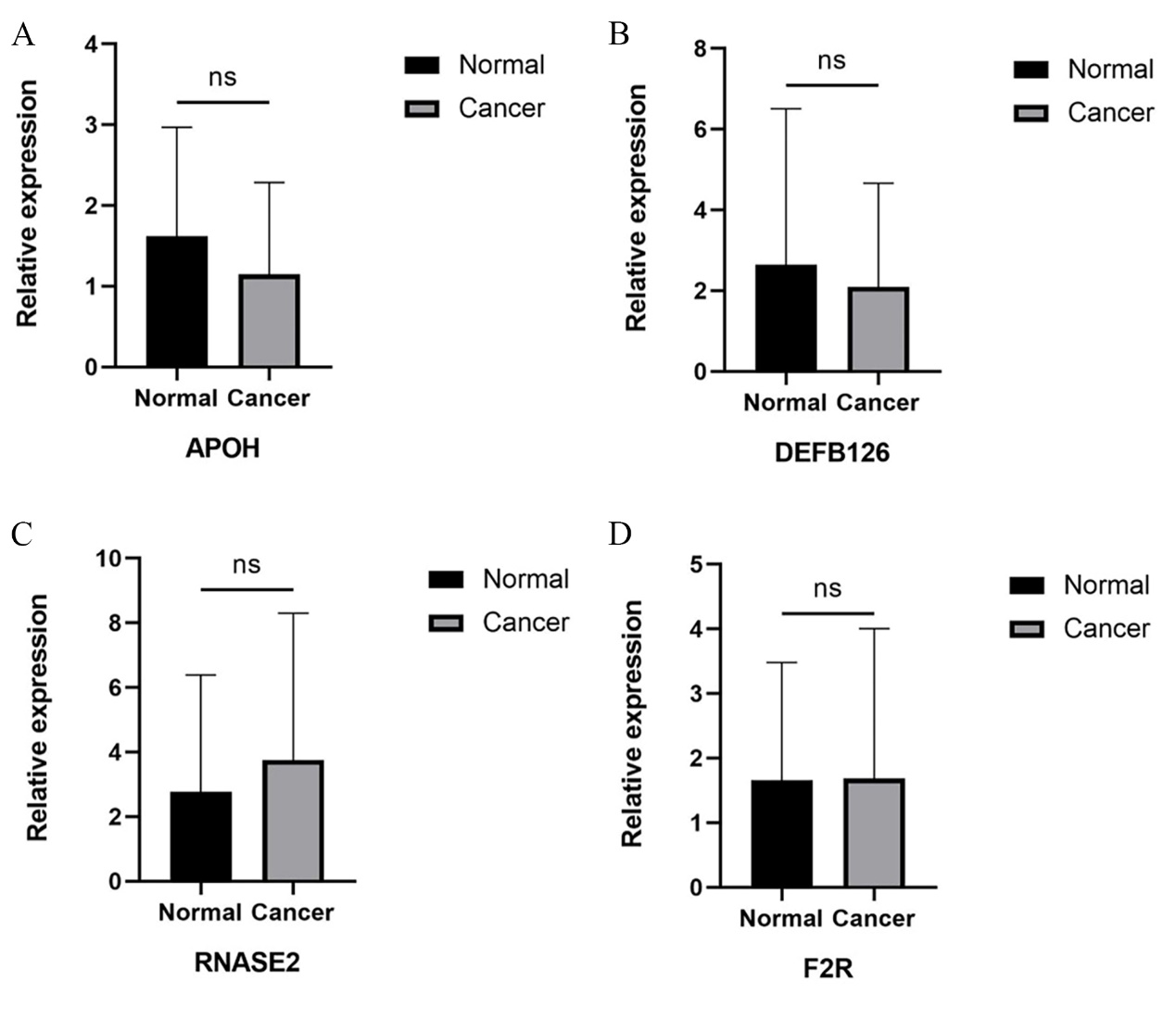


**Supplementary Figure 1 |** The mRNA levels of four immune genes, including APOH, DEFB126, RNASE2, and F2R, in seventeen pairs of GC tissues and their paired adjacent normal tissues were measured by PCR (paired t-test, ns: P ＞ 0.05). (A) APOH (B) DEFB126 (C) RNASE2 (D) F2R.


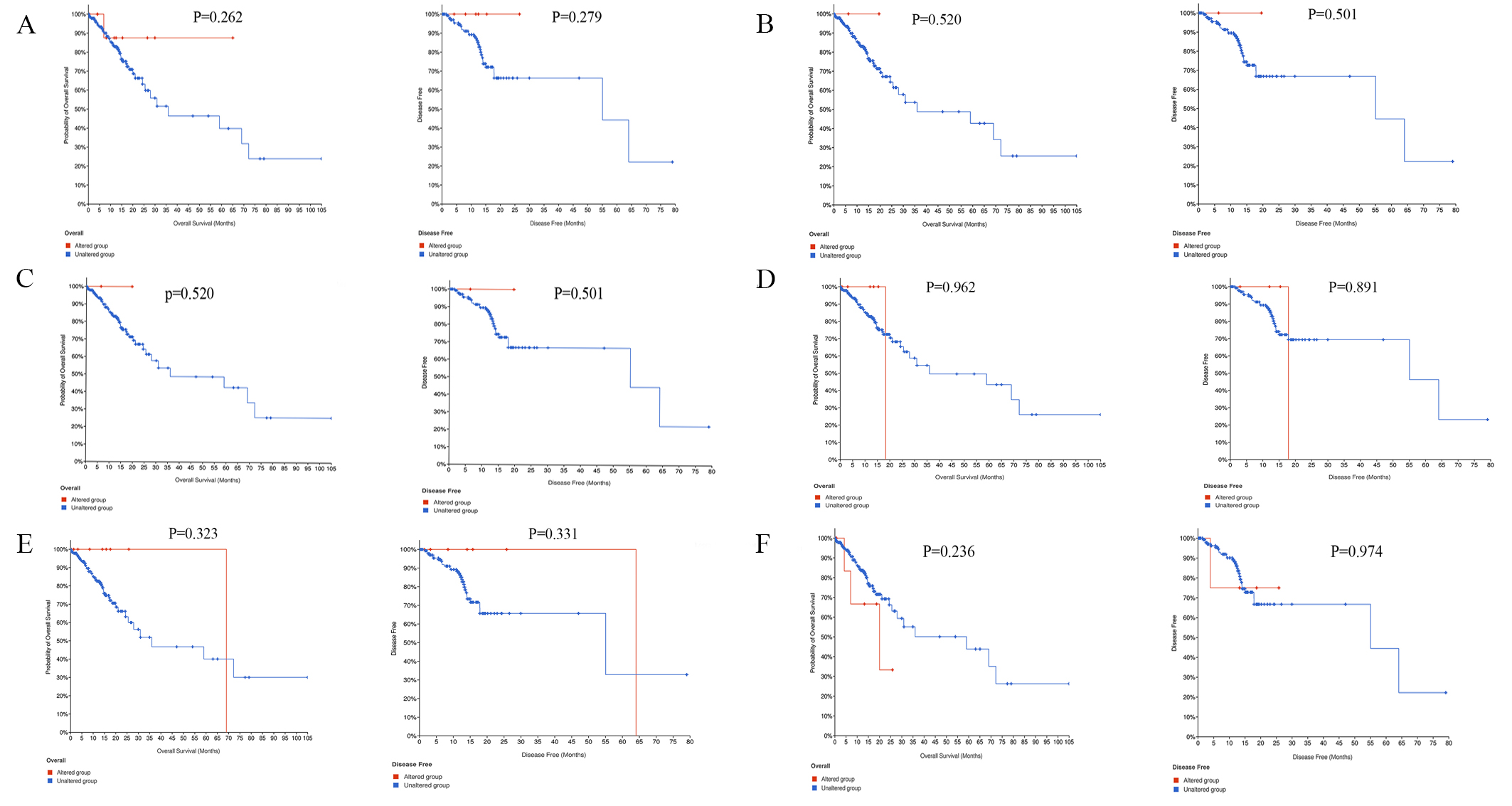


**Supplementary Figure 2 |** Relationships between genetic alterations and survival of GC patients are shown in Figure 2A, B, C, D, E, F: (A) APOH, (B) CXCL3, (C) CXCL6, (D) DEFB126, (E) F2R, and (F) RNASE2.
